# Methyl 2-(3-benzoyl­thio­ureido)acetate

**DOI:** 10.1107/S1600536809046169

**Published:** 2009-11-14

**Authors:** Ibrahim N. Hassan, Bohari M. Yamin, Mohammad B. Kassim

**Affiliations:** a School of Chemical Sciences and Food Technology, Faculty of Science and Technology, Universiti Kebangsaan Malaysia, UKM 43600 Bangi Selangor, Malaysia

## Abstract

In the title compound, C_11_H_12_N_2_O_3_S, the methyl acetate and benzoyl groups adopt a *cis*-*trans* configuration with respect to the thiono S atom across the C—N bonds. An intra­molecular N—H⋯O hydrogen bond is observed. In the crystal packing, mol­ecules are linked by inter­molecular N—H⋯S and C—H⋯O hydrogen bonds to form a two-dimensional network lying parallel to (101).

## Related literature

For bond-length data, see: Allen *et al.* (1987 ▶). For related structures, see: Hassan *et al.* (2008*a* ▶,*b* ▶,*c* ▶); Yamin & Hassan (2004 ▶). For the preparation, see: Hassan *et al.* (2008*a* ▶).
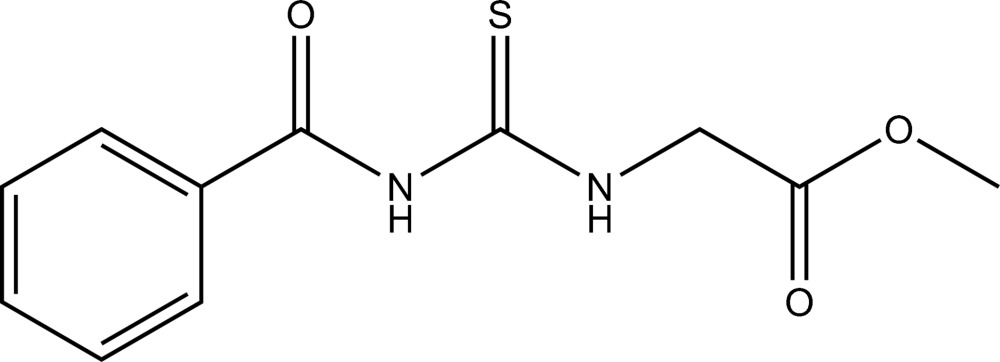


## Experimental

### 

#### Crystal data

C_11_H_12_N_2_O_3_S
*M* *_r_* = 252.29
Monoclinic, 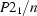

*a* = 14.5804 (15) Å
*b* = 4.9740 (5) Å
*c* = 16.9133 (16) Å
β = 96.210 (2)°
*V* = 1219.4 (2) Å^3^
*Z* = 4
Mo *K*α radiation
μ = 0.26 mm^−1^
*T* = 298 K
0.48 × 0.14 × 0.06 mm

#### Data collection

Bruker SMART APEX CCD area-detector diffractometer
Absorption correction: multi-scan (*SADABS*; Bruker, 2000 ▶) *T*_min_ = 0.884, *T*_max_ = 0.984
6881 measured reflections
2264 independent reflections
1600 reflections with *I* > 2σ(*I*)
*R*_int_ = 0.048

#### Refinement

*R*[*F* ^2^ > 2σ(*F* ^2^)] = 0.089
*wR*(*F* ^2^) = 0.172
*S* = 1.24
2264 reflections
154 parameters
H-atom parameters constrained
Δρ_max_ = 0.26 e Å^−3^
Δρ_min_ = −0.18 e Å^−3^

### 

Data collection: *SMART* (Bruker, 2000 ▶); cell refinement: *SAINT* (Bruker, 2000 ▶); data reduction: *SAINT*; program(s) used to solve structure: *SHELXS97* (Sheldrick, 2008 ▶); program(s) used to refine structure: *SHELXL97* (Sheldrick, 2008 ▶); molecular graphics: *SHELXTL* (Sheldrick, 2008 ▶); software used to prepare material for publication: *SHELXTL*, *PARST* (Nardelli, 1995 ▶) and *PLATON* (Spek, 2009 ▶).

## Supplementary Material

Crystal structure: contains datablocks global, I. DOI: 10.1107/S1600536809046169/ci2952sup1.cif

Structure factors: contains datablocks I. DOI: 10.1107/S1600536809046169/ci2952Isup2.hkl

Additional supplementary materials:  crystallographic information; 3D view; checkCIF report

## Figures and Tables

**Table 1:** Hydrogen-bond geometry (Å, °)

*D*—H⋯*A*	*D*—H	H⋯*A*	*D*⋯*A*	*D*—H⋯*A*
N2—H2*A*⋯O1	0.86	2.02	2.676 (4)	132
N1—H1*A*⋯S1^i^	0.86	2.77	3.547 (3)	151
C9—H9*A*⋯O2^ii^	0.97	2.54	3.358 (6)	142
C9—H9*A*⋯O2^iii^	0.97	2.58	3.211 (5)	123

Symmetry codes: (i) 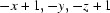; (ii) 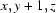; (iii) 
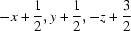
.

**Fig. 1.**
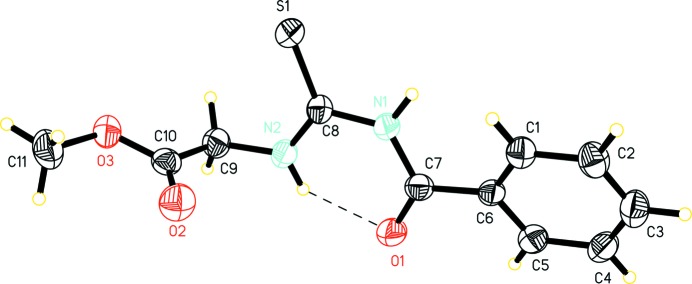
The molecular structure of the title compound, with displacement ellipsoids are drawn at the 50% probability level. The dashed line indicates a hydrogen bond.

**Fig. 2.**
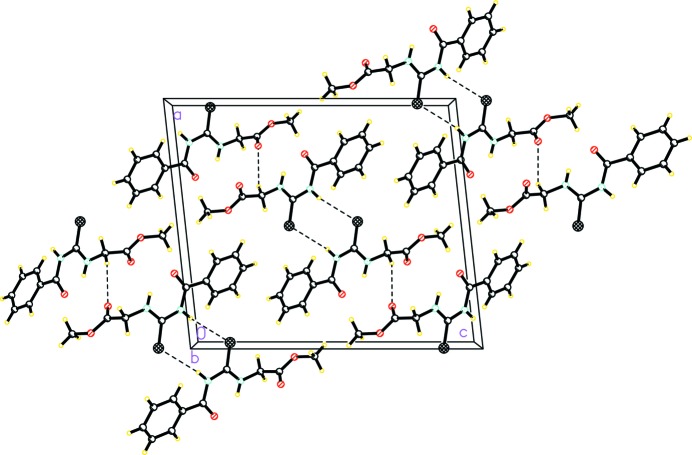
Crystal packing of the title compound, viewed normal to the (101). Hydrogen bonds are shown as dashed lines.

## supplementary crystallographic information

### Comment

The title compound, (I), is a methyl ester derivative of glycine thiourea analogous to our previously reported ethyl-2-(3-benzoylthioureido)acetate (II) (Hassan *et al.*, 2008*a*), propyl-2-(3-benzoylthioureido)acetate (III) (Hassan *et al.*, 2008*b*) and butyl-2-(3-benzoylthioureido)acetate (IV) (Hassan *et al.*, 2008*c*). The methyl acetate fragment and the benzoyl group adopt a *cis*-*trans* configuration, respectively, with respect to the thiono S atom across the C—N bonds (Fig 1). The dihedral angle between the phenyl ring (C1–C6) and the central fragment (S1/N1/N2/C8/C9) is 20.12 (19)°. The bond lengths (Allen *et al.*, 1987) and angles in the molecule are in normal ranges and comparable to those of (II), (III) and (IV). The methyl acetate group (O2/O3/C9/C10/C11) is planar, with a maximum deviation of 0.023 (3) Å for atom O3. The dihedral angle between the phenyl ring and the methyl acetate group is 73.4 (2)°. An intramolecular N2—H2A···O1 hydrogen bond (Table 1) forms a pseudo-five-membered N2/H2A/O1/C7/N1/C8 ring.

Intermolecular N1—H1A···S1 and C9—H9A···O2 hydrogen bonds (Table 1) link the molecules into a two-dimensional network parallel to the (101) (Fig 2).

### Experimental

The title compound was synthesized according to a previously reported method (Hassan *et al.*, 2008*a*). Yellowish crystals, suitable for X-ray analysis, were obtained by slow evaporation of a CH_2_Cl_2_ solution at room temperature (yield 73%).

### Refinement

H atoms were positioned geometrically [N-H = 0.86 Å and C-H = 0.93-0.97 Å] and allowed to ride on their parent atoms, with *U*_iso_(H) = 1.2*U*_eq_ (C,N) and 1.5*U*_eq_(C_methyl_).

### Crystal data

**Table:**

C_11_H_12_N_2_O_3_S	*F*(000) = 528
*M**_r_* = 252.29	*D*_x_ = 1.374 Mg m^−^^3^
Monoclinic, *P*2_1_/*n*	Mo *K*α radiation, λ = 0.71073 Å
Hall symbol: -P 2yn	Cell parameters from 871 reflections
*a* = 14.5804 (15) Å	θ = 1.8–25.5°
*b* = 4.9740 (5) Å	µ = 0.26 mm^−^^1^
*c* = 16.9133 (16) Å	*T* = 298 K
β = 96.210 (2)°	Needle, colourless
*V* = 1219.4 (2) Å^3^	0.48 × 0.14 × 0.06 mm
*Z* = 4	

### Data collection

**Table:**

Bruker SMART APEX CCD area-detector diffractometer	2264 independent reflections
Radiation source: fine-focus sealed tube	1600 reflections with *I* > 2σ(*I*)
graphite	*R*_int_ = 0.048
ω scans	θ_max_ = 25.5°, θ_min_ = 1.8°
Absorption correction: multi-scan (*SADABS*; Bruker, 2000)	*h* = −17→17
*T*_min_ = 0.884, *T*_max_ = 0.984	*k* = −5→6
6881 measured reflections	*l* = −17→20

### Refinement

**Table:**

Refinement on *F*^2^	Primary atom site location: structure-invariant direct methods
Least-squares matrix: full	Secondary atom site location: difference Fourier map
*R*[*F*^2^ > 2σ(*F*^2^)] = 0.089	Hydrogen site location: inferred from neighbouring sites
*wR*(*F*^2^) = 0.172	H-atom parameters constrained
*S* = 1.24	*w* = 1/[σ^2^(*F*_o_^2^) + (0.0474*P*)^2^ + 0.746*P*] where *P* = (*F*_o_^2^ + 2*F*_c_^2^)/3
2264 reflections	(Δ/σ)_max_ = 0.001
154 parameters	Δρ_max_ = 0.26 e Å^−^^3^
0 restraints	Δρ_min_ = −0.18 e Å^−^^3^

### Special details

**Table:**

Geometry. All e.s.d.'s (except the e.s.d. in the dihedral angle between two l.s. planes) are estimated using the full covariance matrix. The cell e.s.d.'s are taken into account individually in the estimation of e.s.d.'s in distances, angles and torsion angles; correlations between e.s.d.'s in cell parameters are only used when they are defined by crystal symmetry. An approximate (isotropic) treatment of cell e.s.d.'s is used for estimating e.s.d.'s involving l.s. planes.
Refinement. Refinement of *F*^2^ against ALL reflections. The weighted *R*-factor *wR* and goodness of fit *S* are based on *F*^2^, conventional *R*-factors *R* are based on *F*, with *F* set to zero for negative *F*^2^. The threshold expression of *F*^2^ > σ(*F*^2^) is used only for calculating *R*-factors(gt) *etc*. and is not relevant to the choice of reflections for refinement. *R*-factors based on *F*^2^ are statistically about twice as large as those based on *F*, and *R*- factors based on ALL data will be even larger.

### Fractional atomic coordinates and isotropic or equivalent isotropic displacement parameters (Å^2^)

**Table:**

	*x*	*y*	*z*	*U*_iso_*/*U*_eq_	
S1	0.50901 (8)	0.0880 (3)	0.62465 (7)	0.0818 (5)	
O1	0.2035 (2)	0.1002 (7)	0.53998 (17)	0.0725 (10)	
N1	0.3541 (2)	−0.0034 (7)	0.53458 (18)	0.0545 (9)	
H1A	0.3909	−0.0822	0.5055	0.065*	
C9	0.3780 (3)	0.4046 (9)	0.7161 (2)	0.0610 (12)	
H9A	0.3380	0.5543	0.7257	0.073*	
H9B	0.4388	0.4763	0.7105	0.073*	
C6	0.2391 (3)	−0.2034 (9)	0.4391 (2)	0.0494 (10)	
N2	0.3429 (2)	0.2733 (8)	0.64340 (18)	0.0576 (10)	
H2A	0.2854	0.2906	0.6265	0.069*	
O3	0.4423 (2)	0.3093 (7)	0.84511 (17)	0.0740 (10)	
C7	0.2621 (3)	−0.0237 (9)	0.5083 (2)	0.0535 (11)	
C1	0.2954 (3)	−0.4110 (9)	0.4191 (2)	0.0555 (11)	
H1B	0.3509	−0.4435	0.4501	0.067*	
O2	0.3394 (2)	0.0172 (7)	0.7891 (2)	0.0812 (10)	
C10	0.3840 (3)	0.2182 (10)	0.7862 (2)	0.0553 (11)	
C8	0.3961 (3)	0.1265 (9)	0.6015 (2)	0.0542 (11)	
C5	0.1559 (3)	−0.1619 (9)	0.3927 (2)	0.0602 (12)	
H5A	0.1168	−0.0250	0.4056	0.072*	
C4	0.1308 (3)	−0.3236 (10)	0.3269 (3)	0.0654 (13)	
H4A	0.0753	−0.2938	0.2956	0.078*	
C3	0.1877 (4)	−0.5258 (11)	0.3085 (3)	0.0704 (14)	
H3A	0.1707	−0.6352	0.2647	0.084*	
C2	0.2700 (3)	−0.5688 (10)	0.3542 (3)	0.0640 (13)	
H2B	0.3088	−0.7062	0.3409	0.077*	
C11	0.4497 (4)	0.1530 (13)	0.9180 (3)	0.0918 (19)	
H11A	0.4937	0.2362	0.9568	0.138*	
H11B	0.4698	−0.0259	0.9074	0.138*	
H11C	0.3906	0.1454	0.9381	0.138*	

### Atomic displacement parameters (Å^2^)

**Table:**

	*U*^11^	*U*^22^	*U*^33^	*U*^12^	*U*^13^	*U*^23^
S1	0.0492 (7)	0.1335 (14)	0.0612 (8)	0.0052 (8)	−0.0012 (5)	−0.0188 (8)
O1	0.0499 (18)	0.093 (3)	0.075 (2)	0.0001 (17)	0.0082 (15)	−0.0248 (19)
N1	0.0436 (19)	0.070 (3)	0.049 (2)	0.0069 (17)	0.0017 (15)	−0.0076 (18)
C9	0.065 (3)	0.059 (3)	0.058 (3)	−0.006 (2)	0.006 (2)	−0.009 (2)
C6	0.049 (2)	0.055 (3)	0.045 (2)	−0.014 (2)	0.0068 (19)	0.003 (2)
N2	0.052 (2)	0.066 (3)	0.054 (2)	0.0030 (19)	−0.0007 (17)	−0.0090 (19)
O3	0.073 (2)	0.095 (3)	0.0531 (18)	−0.0153 (19)	0.0021 (15)	−0.0035 (18)
C7	0.048 (2)	0.058 (3)	0.055 (3)	−0.002 (2)	0.008 (2)	0.002 (2)
C1	0.062 (3)	0.051 (3)	0.054 (3)	−0.004 (2)	0.005 (2)	0.001 (2)
O2	0.092 (2)	0.065 (2)	0.086 (2)	−0.017 (2)	0.0060 (18)	0.0004 (19)
C10	0.051 (3)	0.056 (3)	0.060 (3)	0.001 (2)	0.014 (2)	−0.009 (2)
C8	0.054 (3)	0.062 (3)	0.045 (2)	−0.001 (2)	0.0012 (19)	0.001 (2)
C5	0.058 (3)	0.060 (3)	0.063 (3)	−0.007 (2)	0.008 (2)	−0.003 (2)
C4	0.059 (3)	0.075 (4)	0.059 (3)	−0.016 (3)	−0.006 (2)	0.006 (3)
C3	0.093 (4)	0.063 (3)	0.054 (3)	−0.018 (3)	0.005 (3)	−0.004 (3)
C2	0.080 (3)	0.051 (3)	0.062 (3)	0.000 (2)	0.011 (2)	−0.005 (2)
C11	0.098 (4)	0.123 (5)	0.055 (3)	0.017 (4)	0.008 (3)	0.011 (3)

### Geometric parameters (Å, °)

**Table:**

S1—C8	1.661 (4)	O3—C11	1.452 (5)
O1—C7	1.223 (5)	C1—C2	1.368 (6)
N1—C7	1.371 (5)	C1—H1B	0.93
N1—C8	1.387 (5)	O2—C10	1.197 (5)
N1—H1A	0.86	C5—C4	1.389 (6)
C9—N2	1.437 (5)	C5—H5A	0.93
C9—C10	1.499 (6)	C4—C3	1.361 (6)
C9—H9A	0.97	C4—H4A	0.93
C9—H9B	0.97	C3—C2	1.372 (6)
C6—C1	1.384 (6)	C3—H3A	0.93
C6—C5	1.386 (5)	C2—H2B	0.93
C6—C7	1.482 (6)	C11—H11A	0.96
N2—C8	1.326 (5)	C11—H11B	0.96
N2—H2A	0.86	C11—H11C	0.96
O3—C10	1.318 (5)		
			
C7—N1—C8	129.0 (3)	O2—C10—C9	124.3 (4)
C7—N1—H1A	115.5	O3—C10—C9	111.3 (4)
C8—N1—H1A	115.5	N2—C8—N1	117.4 (4)
N2—C9—C10	112.4 (4)	N2—C8—S1	124.0 (3)
N2—C9—H9A	109.1	N1—C8—S1	118.6 (3)
C10—C9—H9A	109.1	C6—C5—C4	120.4 (4)
N2—C9—H9B	109.1	C6—C5—H5A	119.8
C10—C9—H9B	109.1	C4—C5—H5A	119.8
H9A—C9—H9B	107.9	C3—C4—C5	119.8 (4)
C1—C6—C5	118.6 (4)	C3—C4—H4A	120.1
C1—C6—C7	123.6 (4)	C5—C4—H4A	120.1
C5—C6—C7	117.9 (4)	C4—C3—C2	120.2 (5)
C8—N2—C9	122.3 (4)	C4—C3—H3A	119.9
C8—N2—H2A	118.9	C2—C3—H3A	119.9
C9—N2—H2A	118.9	C1—C2—C3	120.5 (5)
C10—O3—C11	116.2 (4)	C1—C2—H2B	119.8
O1—C7—N1	121.8 (4)	C3—C2—H2B	119.8
O1—C7—C6	122.7 (4)	O3—C11—H11A	109.5
N1—C7—C6	115.5 (4)	O3—C11—H11B	109.5
C2—C1—C6	120.5 (4)	H11A—C11—H11B	109.5
C2—C1—H1B	119.7	O3—C11—H11C	109.5
C6—C1—H1B	119.7	H11A—C11—H11C	109.5
O2—C10—O3	124.4 (4)	H11B—C11—H11C	109.5

### Hydrogen-bond geometry (Å, °)

**Table:**

*D*—H···*A*	*D*—H	H···*A*	*D*···*A*	*D*—H···*A*
N2—H2A···O1	0.86	2.02	2.676 (4)	132
C9—H9B···S1	0.97	2.68	3.027 (4)	101
N1—H1A···S1^i^	0.86	2.77	3.547 (3)	151
C9—H9A···O2^ii^	0.97	2.54	3.358 (6)	142
C9—H9A···O2^iii^	0.97	2.58	3.211 (5)	123

Symmetry codes: (i) −*x*+1, −*y*, −*z*+1; (ii) *x*, *y*+1, *z*; (iii) −*x*+1/2, *y*+1/2, −*z*+3/2.
